# Pyrolyzed Parylene Electrodes for Detection of Tryptophan, Tyrosine, and Gonadotropin-Releasing Hormone

**DOI:** 10.1021/acsmeasuresciau.5c00165

**Published:** 2025-12-26

**Authors:** Faith Eyimegwu, He Zhao, Kailash Shrestha, Dayana Surendran, Nickolay V. Lavrik, B. Jill Venton

**Affiliations:** † Department of Chemistry, 2358 University of Virginia, Charlottesville, Virginia 22901, United States; ‡ Center for Nanophase Materials Sciences, 6146 Oak Ridge National Lab, Oak Ridge, Tennessee 37831, United States

**Keywords:** carbon electrodes, adsorption, parylene, RTP, GnRH

## Abstract

Sensitive and selective detection of neurochemicals such as neuropeptides is critical for understanding brain signaling. While carbon-fiber microelectrodes (CFMEs) are widely used for these measurements, alternative electrode materials and fabrication techniques could improve sensitivity and versatility. In this study, we investigate pyrolyzed parylene-N microelectrodes (PPNMEs) as a promising platform for making thin-film carbon electrodes for the detection of electroactive amino acids and neuropeptides. We evaluated the performance of PPNMEs for the detection of tryptophan (Trp), tyrosine (Tyr), and the neuropeptide gonadotropin-releasing hormone (GnRH), which contains these electroactive residues. PPNMEs demonstrated significantly greater sensitivity with fast-scan cyclic voltammetry, with signal amplitudes approximately four times higher than those observed with CFMEs. After normalization for surface area, PPNMEs exhibited 3-, 5-, and 2.7-fold higher signals than CFMEs for Trp, Tyr, and GnRH, respectively. Additionally, PPNMEs facilitated faster electron transfer kinetics, as evidenced by reduced oxidation potentials. There were enhanced signals for secondary oxidation peaks at PPNMEs because the rougher surface can trap intermediates near the surface, facilitating detection of downstream electrochemical reactions. Scan rate analysis indicates more adsorption-controlled detection, contributing to improved sensitivity. Importantly, PPNMEs enabled sensitive detection of GnRH in brain tissue slices, including both puffed-on applications and spontaneous endogenous GnRH release in the median eminence. These results highlight the potential of PPNMEs as a new class of carbon-based electrodes, offering a promising alternative to CFMEs for high-sensitivity, low-potential detection of neurochemicals in biological tissues.

## Graphical abstract

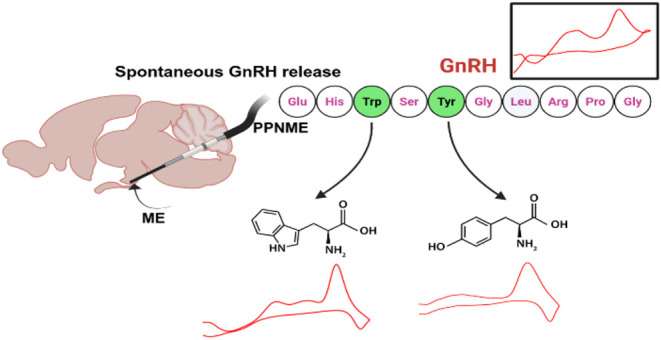

## Introduction

Carbon-fiber microelectrodes (CFMEs) are widely used for the electrochemical detection of biomolecules via fast-scan cyclic voltammetry (FSCV) due to their active surface area, excellent conductivity, and biocompatibility.^1,2^ Different carbon-based materials have also been incorporated into electrode designs; for example, carbon nanotubes (CNTs) can be coated onto electrodes or fabricated into fibers, such as CNT yarns (CNTYs),^3–5^ while carbon nanohorns,^6^ carbon nanofibers^7^ and forms of graphene have also been utilized to enhance electron transfer rates and sensitivity.^8,9^ Beyond these materials, recent advances in electrode fabrication have enabled the development of new electrodes using alternative carbon sources.

Parylene-N (PPN) can be conformally deposited onto substrates via chemical vapor deposition (CVD) and then pyrolyzed, resulting in thin carbon films with a high density of edge-plane sites and tunable surface chemistry.^10,11^ Parylene, or poly­(*p*-xylene), is a benzene-rich polymer valued for its chemical inertness, flexibility, and transparency, and is commonly used as an insulating material in electronics.^11,12^ After pyrolysis to graphene, pyrolyzed parylene films enhanced electrochemical performance compared to traditional CFMEs for dopamine.^10^ In particular, adding a hot plate activation step increases the density of states (DOS) and promotes neurotransmitter adsorption, amplifying sensitivity.^10^ Unlike traditional carbon fibers, PPN microelectrodes can be fabricated in different geometries and are compatible with emerging methods, such as laser-induced graphene (LIG), which opens new possibilities for electrode design and integration.^13^ PPNMEs have enhanced performance for dopamine detection with fast-scan cyclic voltammetry (FSCV),^10^ but they have not been characterized for a broad range of neurochemicals, including amino acids and neuropeptides.

While neurochemical studies have traditionally focused on monoamine neurotransmitters, such as dopamine and serotonin,^10^ there is growing interest in extending these methods to other electroactive species, including amino acids like tryptophan and tyrosine, as well as neuropeptides that contain gonadotropin-releasing hormone (GnRH).^14^ Tryptophan (Trp) is a nonpolar, aromatic amino acid with an indole side chain and serves as a precursor for several neurohormones, most notably melatonin and serotonin.^15,16^ The electrochemical oxidation of tryptophan is well characterized, involving a two-electron process that eliminates the pyrrole double bond and adds a ketone adjacent to the nitrogen atom, followed by further possible oxidation to multiple products (Scheme 1).^17,18^ Tyrosine (Tyr), another aromatic amino acid with a phenolic side chain, is a biosynthetic precursor to catecholamine neurotransmitters, including dopamine, epinephrine, and norepinephrine.^19^ Tyrosine has been employed as a marker for quantifying small neuropeptides released in the opioid pathway via FSCV.^20,21^ Its primary and secondary oxidation potentials are typically observed at approximately 1.13 and 0.39 V, respectively.^22^ Neuropeptides containing electroactive amino acids such as Trp and Tyr are electrochemically detected. Sombers’ group has pioneered the use of FSCV for detecting neuropeptides, including enkephalins, in the brain.^20,23^ These studies demonstrate the feasibility of monitoring transient neuropeptide release events in real time and underscore the value of electrochemical techniques for neurochemical analysis.

**Scheme 1:**
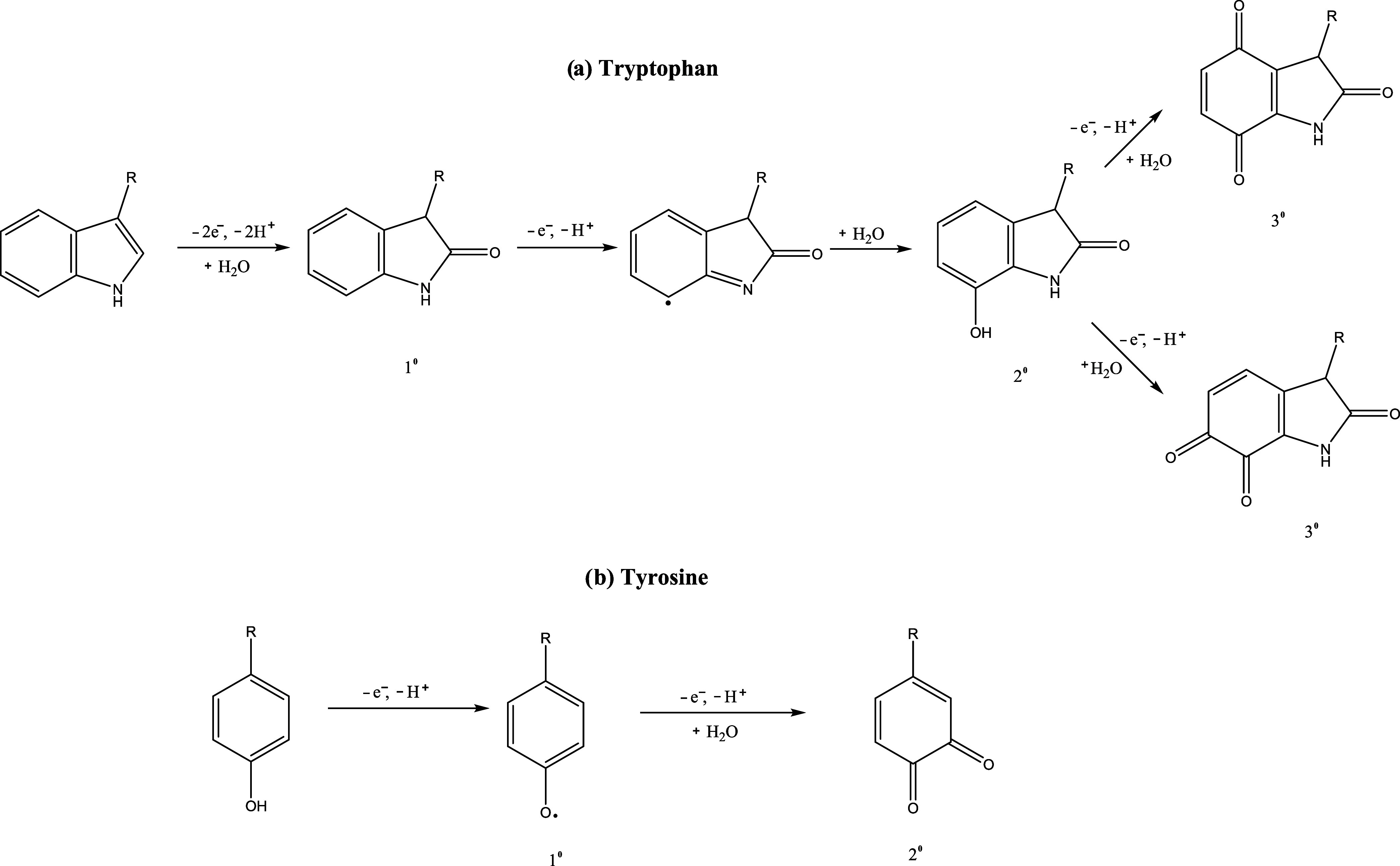
(A) Proposed Oxidative Mechanism of Tryptophan; Abbreviated R is the Amino Acid Backbone. (B) Proposed Oxidative Mechanism of Tyrosine, Abbreviated R, is the Amino Acid Backbone

Our group has measured transient release of GnRH in brain slices, further expanding the application of FSCV to neuropeptides beyond the opioid pathway.^14^ Gonadotropin-releasing hormone (GnRH) is a neuropeptide produced in the hypothalamus that plays a pivotal role in regulating reproductive function by stimulating the anterior pituitary to synthesize and secrete luteinizing hormone (LH) and follicle-stimulating hormone (FSH).^24–26^ Accurate measurement of GnRH is essential for advancing our understanding of reproductive physiology and diagnosing related disorders. Notably, GnRH contains electroactive amino acids, specifically tryptophan and tyrosine, which can be detected and characterized by FSCV based on their redox properties.

The objective of this study is to systematically evaluate the electrochemical behavior of tryptophan, tyrosine, and GnRH at pyrolyzed parylene-N modified electrodes (PPNMEs) and compare their performance to CFMEs. We found that PPNMEs offered enhanced adsorption, increased sensitivity, and lower oxidation potentials for tryptophan compared to CFMEs. Tyrosine detection is similarly improved, exhibiting a combination of diffusion- and adsorption-controlled processes, along with a shift to lower oxidation potentials. PPNMEs exhibited enhanced sensitivity for GnRH and were used to detect GnRH in tissue, including both puffed-on application and spontaneous endogenous GnRH release in the median eminence. These findings indicate that PPN-modified electrodes are highly effective for precise measurement of neuropeptides and electroactive amino acids and hold significant promise for advancing research in reproductive physiology and neurochemistry.

## Experimental Methods

### Chemicals and Materials

Tryptophan, tyrosine, and gonadotropin-releasing hormone (GnRH) were obtained from Sigma-Aldrich (St. Louis, MO). Stock solutions (10 mM) were prepared in 0.1 M HClO_4_ and subsequently diluted to working concentrations (1 μM) using phosphate-buffered saline (PBS; 131.25 mM NaCl, 3.00 mM KCl, 10 mM NaH_2_PO_4_, 1.2 mM MgCl_2_, 2.0 mM Na_2_SO_4_, and 1.2 mM CaCl_2_, pH 7.4). All aqueous solutions were prepared with deionized water (EMD Millipore, Billerica, MA).

### Preparation of PPNMEs

Niobium wires (50 μm diameter, Advent Research Materials, Eynsham, Oxford) were electrochemically etched to a tip diameter of 1 μm in 4 M NaOH at 2 V DC for 10 min. The etched wires were coated with parylene-N (PN) using a chemical vapor deposition system (SCS, Indianapolis, IN). Dipara-xylene powder, used as a precursor, was vaporized in the parylene coating chamber at 150 °C under vacuum conditions. The dimer was then subjected to pyrolysis at 650 °C, breaking it down into the monomer para-xylene, which subsequently polymerized to form the poly­(para-xylene) structure known as parylene. The PN-coated wires were preannealed on a micro hot plate at 350 °C for 10 min in the air. Carbonization was performed in a rapid thermal processor (RTP, First nano, NY) in two stages: first at 600 °C in argon (9 Torr, 10 min), then at 950 °C in argon (1 Torr, 10 min), yielding pyrolyzed parylene-N. The resulting wires were insulated with glass capillaries, exposing 100 μm of the electrode tip, and sealed with epoxy for 5 min (J-B Weld, Sulfur Springs, TX).

Niobium wire served only as an insulated current collector. Bare niobium does not produce detectable faradaic currents for neurotransmitters under FSCV conditions, as demonstrated in previous studies where carbon-coated niobium wires exhibited electrochemical activity while uncoated niobium did not.^27^ Thus, all electrochemical responses reported here originate from the pyrolyzed parylene-N (RTP-PN) carbon film at the electrode tip.

The electrode was not subjected to separate electrochemical cleaning or conditioning steps between calibration points. During the analytical curve measurements, consecutive CV scans produced highly reproducible baseline-subtracted currents, and no drift in peak position was observed. This confirmed that the electrode surface remained stable throughout the calibration sequence, making extra conditioning unnecessary.

### Preparation of CFMEs

Seven μm diameter Carbon fibers (T650–35, Cytec, Woodland Park, NJ) were inserted into borosilicate glass capillaries. The capillaries were pulled using a vertical thermal puller (Setagaya-ku, Tokyo, Japan) to form two microelectrodes per capillary. The exposed fiber length was trimmed to 50–100 μm. Electrodes were dipped for 30 s in a mixture of Epon Resin 828 (Danbury, CT) with 14% (w/w) *m*-phenylenediamine hardener (Acros Organics, Morris Plains, NJ) to seal the gap between the glass-fiber interface, and then rinsed in acetone for 5 s to remove excess epoxy, air-dried overnight, and cured at 100 °C for 2 h and 150 °C overnight.

### Electrochemical Measurements

FSCV was performed using a ChemClamp potentiostat (Dagan, Minneapolis, MN) with a 1 MΩ headstage. The applied waveform ranged from −0.4 to 1.3 V at a scan rate of 400 V/s and a frequency of 10 Hz. Data was acquired and analyzed using HDCV Analysis Software (University of North Carolina at Chapel Hill). A silver/silver chloride wire served as the reference electrode. Flow injection analysis was performed using a six-port stainless steel HPLC loop injector (VICI Valco Instruments, Houston, TX) with a dual syringe pump operating at a flow rate of 2 mL/min. The headstage was connected via a glass capillary filled with 4 M KCl and a silver wire (Warner Instruments, Holliston, MA).

Because geometric surface area severely underestimates the true electroactive surface of porous carbon materials, we did not calculate surface area using geometric or amperometric methods. Instead, the background charging current obtained from the FSCV waveform was used as an electrochemical proxy for effective surface area (Figure S1). Faradaic currents were normalized to the corresponding background current to allow accurate comparison between electrode types.

### Brain Slice Puffed-on GnRH

All animal procedures were approved by the Animal Care and Use Committee at the University of Virginia. Wild-type C57BL/6 mice (5–8 weeks old) were anesthetized with isoflurane and decapitated. Brains were quickly removed and placed in ice-cold, oxygenated artificial cerebrospinal fluid (aCSF; 95% O_2_, 5% CO_2_). Coronal brain slices (400 μm thick) containing the caudate putamen were prepared using a vibratome (Leica VT1000S) and then equilibrated in oxygenated aCSF at 34 °C. Slices were transferred to a recording chamber and perfused with oxygenated aCSF at a rate of 2 mL/min. PPNMEs were inserted approximately 75 μm into the tissue and allowed to equilibrate for 10–15 min. GnRH was loaded into a glass capillary near the electrode and delivered using a nanoliter injector (Nanoliter2020, World Precision Instruments, FL). Cyclic voltammetry was recorded with HDCV software.

### Brain Slice Spontaneous GnRH release

All experiments were approved by the Animal Care and Use Committee of the University of Virginia. Mice were group-housed and maintained a 12–12-h day-night cycle with ad libitum access to food and water. Wild-type C57BL/6 male mice (8–10 weeks old) were used for FSCV experiments to record spontaneous GnRH release. Mice were anesthetized with isoflurane and decapitated. Brains were quickly removed and placed in ice-cold, oxygenated (95% O_2_, 5% CO_2_) artificial cerebrospinal fluid (aCSF) containing (in mM) 2.5 KCl, 0.5 CaCl_2_, 25 NaHCO_3_, 1 NaH_2_PO_4_, 7 MgCl_2_.6H_2_O, 110 Choline Chloride, and 25 Dextrose (pH −7.4). The median eminence (ME) is a single, symmetrical structure centered precisely on the midline of the brain located −1.6 to −2.0 mm posterior to bregma in the anterior-posterior (AP) plane, according to the Paxinos and Franklin stereotaxic atlas. The brains were cut along the midline sagittal plane to separate the two hemispheres. Then horizontal cuts were made across the cerebral cortex to create a flat dorsal surface, aiding in stable mounting of the hemispheres with the medial surface facing the vibratome (Leica VT1000S) blade. Parasagittal sections of 300 μm thickness were cut and slices containing the median eminence (ME) were collected and transferred to prewarmed (32 °C) oxygen-saturated Ringer’s buffer (RB) containing (in mM) 125 NaCl, 1 NaH_2_PO_4_, 1.3 MgCl_2_.6H_2_O, 2 CaCl_2_, 25 Dextrose, 2.5 KCl, 25 NaHCO_3_ (pH −7.4). The slices were incubated at 32 °C for 30 min to allow metabolic recovery and subsequently kept at room temperature throughout the entire duration of the experiment. Slices were then transferred to a recording chamber mounted on the stage of an upright microscope (Olympus BX51WI). The chamber was perfused with (2 mL/min) with oxygenated RB at a rate of 2 mL/min at 32 °C. PPNMEs were equilibrated in RB by applying a dopamine waveform (−0.4 V, 1.3 V, 400 V/s scan rate at 10 Hz) at the electrode using a WaveNeuro FSCV potentiostat (Pine Research Instrument, NC) and HDCV software (UNC Chemistry Department). PPNMEs were inserted 50–75 μm into the ME and allowed to stabilize for 15 min before recording data. FSCV signals generated from spontaneous GnRH release were recorded.

### Statistical Analysis

Data are presented as mean ± standard error of the mean (SEM) for n electrodes. Each experiment was repeated three times per electrode. Statistical analyses were performed using GraphPad Prism (GraphPad Software, San Diego, CA). The high scan rate of FSCV (400 V/s) generates substantial background currents, making the Faradaic current difficult to detect. To address this, background subtraction is performed during neurochemical detection, but minor errors can occur in the final CV graphs due to analyte adsorption on the electrode surface.

## Results

### Surface Characterization

First, the surfaces of PPNME and CFME were examined using scanning electron microscopy. Figure 1A illustrates the overall morphology of the CF microelectrode, revealing a relatively smooth surface with some visible grooves. The carbon fibers measure approximately 7 μm in diameter. Figure 1B displays the RTP-PN-modified Nb wire. The PPNME formed on etched Nb wire (with 6 g of material deposited) has an overall diameter of approximately 1 μm, significantly smaller than CFs. Thus, the smaller diameter PPNMEs could be more precisely targeted to specific brain regions to minimize tissue inflammation. Additionally, the nanopores in PPNME are both larger and deeper than the surface grooves found on CFMEs, leading to trapping effects that increased sensitivity for dopamine in previous studies.^10^ The surface chemistry of PPNMEs, including oxygen functional group content, has been previously characterized under identical fabrication conditions.^10^ The surface functional groups are C=C (58%), C–O (35%), and C=O (7%) for PPNMEs and C=C (67%), C–O (30%), C=O (3%) for CFMEs, showing that PPNMEs have slightly higher oxygen groups that cause more adsorption of the peptides.

**Figure 1:**
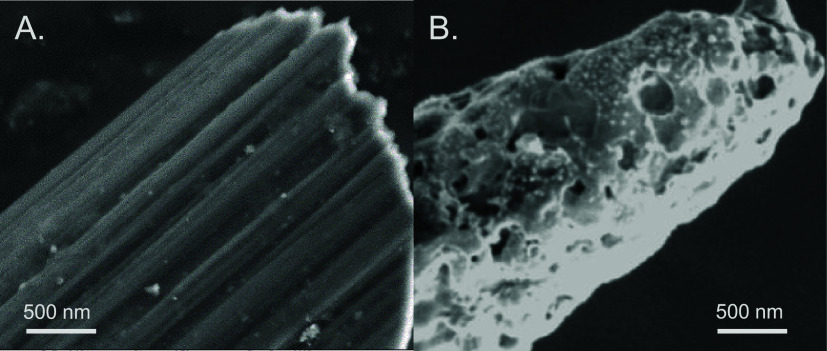
SEM images of the (A) surface of CF and the (B) surface of RTP-PN.

### Electrochemical Detection of Tryptophan

Tryptophan (Trp) is an essential aromatic amino acid, and its electrochemical behavior was compared at CFMEs and PPNMEs. We first recorded cyclic voltammograms (CVs) for 10 μM Trp using the standard “dopamine” waveform (−0.4 to 1.3 V, scan rate 400 V/s, 10 Hz). As shown in Figure 2A, both electrode types produced distinct oxidation peaks, but with notable differences in the number and magnitude of peaks. At the CFME, two oxidation peaks are observed: a primary peak at 1.08 V (55 nA) and a secondary peak at 0.45 V (19 nA). In contrast, tryptophan exhibits three oxidation peaks at PPNMEs: 0.96 V (150 nA), 0.4 V (50 nA), and 0.75 V (49 nA). These peaks align with the established oxidation pattern of Trp, which entails electron and proton loss followed by hydration, resulting in the formation of a carbonyl group on the indole ring. The lower oxidation potentials and substantially higher peak currents at PPNMEs suggest that these electrodes facilitate more rapid electron transfer and provide greater sensitivity for Trp detection compared to CFMEs. This enhanced sensitivity is likely due to the increased surface area and enhanced electron kinetics of the RTP-PN coating,^10^ which can promote adsorption and electron transfer for aromatic amino acids, such as Trp.

**Figure 2:**
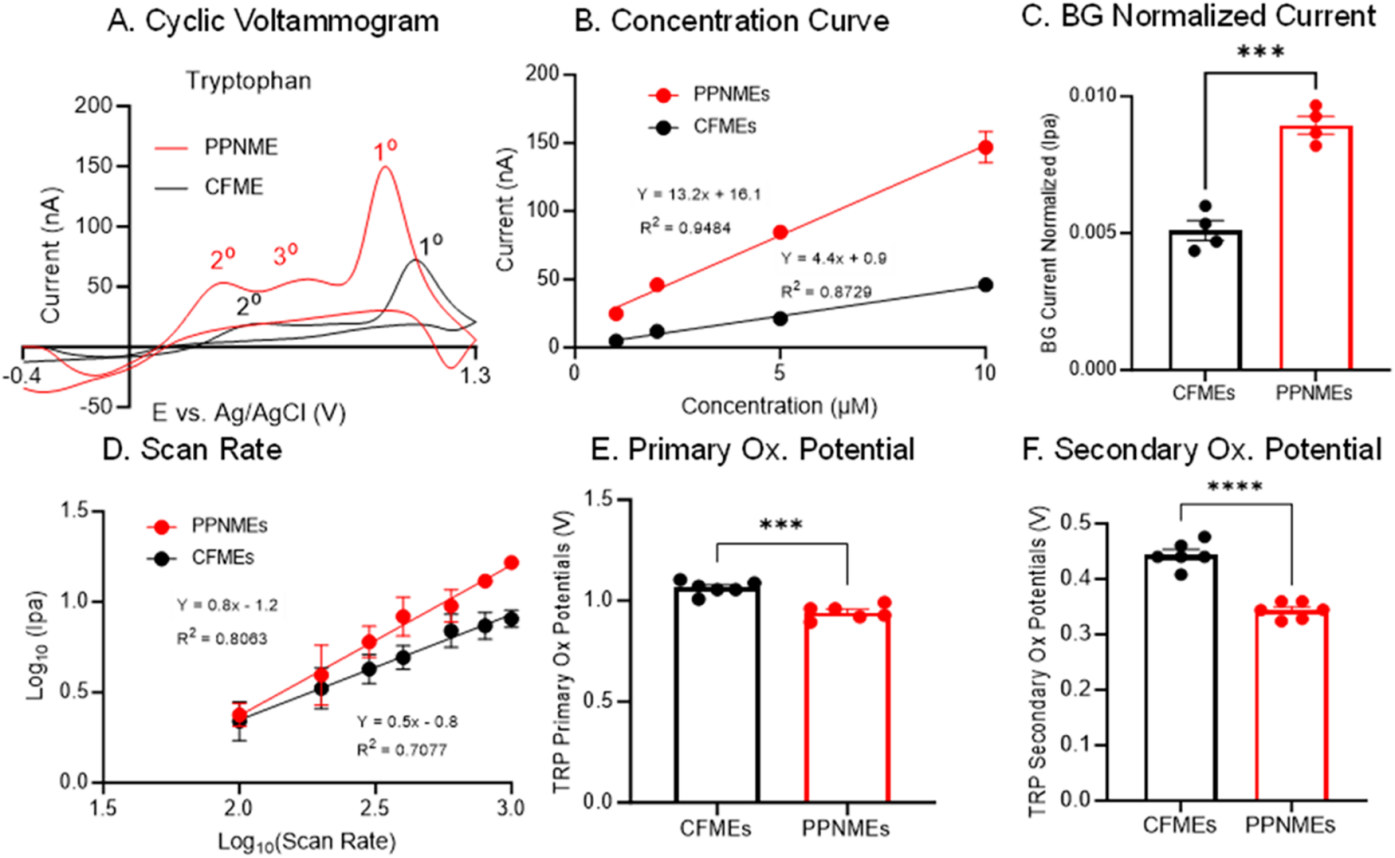
Electrochemical characterization of L-tryptophan at CFMEs and PPNMEs. (A) CVs of 10 μM Trp. (B) Trp sensitivity tests (1–10 μM). The PPNME showed good reproducibility, with RSDs of 6.3% at 5 μM Trp and 15.8% for CFME. (C) Background normalized current comparison for 1 μM Trp (*n* = 4, *t* test, ***p = 0.0002). (D) Trp scan rate tests (100–1000 V/s). (E) Trp primary oxidation potentials (*n* = 6, *t* test, ***p = 0.0001). (F) Trp secondary oxidation potentials (*n* = 6, *t* test, ****p < 0.0001). (Error bars are SEM).

To quantitatively assess sensitivity and linearity, we obtained calibration curves by measuring current responses to increasing Trp concentrations (1–10 μM; Figure 2B). Both electrodes showed linear responses within this range, but the sensitivity (slope) at PPNMEs (13.2 ± 0.8 nA/μM) was over three times higher than at CFMEs (4.4 ± 0.4 nA/μM). This dramatic increase in sensitivity at PPNMEs is likely due to a combination of factors: increased surface roughness, which increases area and adsorption sites.^10,12^

A significantly larger porous surface leads to increased background charging currents (Figure S1). To account for differences in surface area and background charging currents, we normalized the faradaic currents to the background charging current (Figure 2C). Even after normalization, PPNMEs showed significantly higher signals for Trp, confirming that their superior sensitivity is not solely due to increased surface area but to a more active surface. To further probe the mechanism of Trp detection, we performed scan rate studies (Figure 2D). In this log–log plot, a slope of 0.5 is indicative of diffusion-controlled kinetics, and a slope of 1.0 is indicative of adsorption-controlled kinetics. For CFMEs, the slope of the log­(current) vs log (scan rate) plot was 0.58, consistent with a more diffusion-controlled process. In contrast, PPNMEs showed a higher slope of 0.83, indicating a more adsorption-controlled process. The higher slope may also be due to thin-layer diffusion effects, as tryptophan is trapped in the cracks and may not diffuse out on the time scale of the FSCV experiment, thereby preconcentrating Trp.^27^

Finally, we compared the oxidation potentials of Trp at both electrode types (Figure 2E,F). PPNMEs showed lower average primary and secondary oxidation potentials than CFMEs, reflecting an electrocatalytic effect of the PPNME surface. As shown in Figure 2B, reproducibility across four electrodes (*n* = 4) demonstrated low variability (6.29% RSD) in both oxidative peak currents and calibration slopes. Error bars represent SD across electrodes.

### Electrochemical Detection of Tyrosine

We next examined the electrochemical detection of tyrosine (Tyr), another aromatic amino acid, to determine whether the advantages observed for Trp at PPNMEs would extend to other structurally similar analytes. CVs were recorded for 10 μM Tyr at both CFMEs and PPNMEs using the same waveform. At a CFME, Tyr produces two oxidation peaks: a primary peak at 1.02 V and a secondary peak at 0.30 V (Figure 3A). These peaks are consistent with the known oxidation behavior of Tyr, which involves the loss of electrons from the phenolic side chain (Scheme 1b). When measured at PPNMEs, the primary oxidation peak for Tyr slightly shifted to a lower potential (0.89 V), and the peak current increased substantially. The secondary peak also shifted to a lower potential. This shift to lower potential and increase in current suggest that, as with Trp, the PPNME surface facilitates more rapid electron transfer and enhances the oxidation of Tyr.

**Figure 3:**
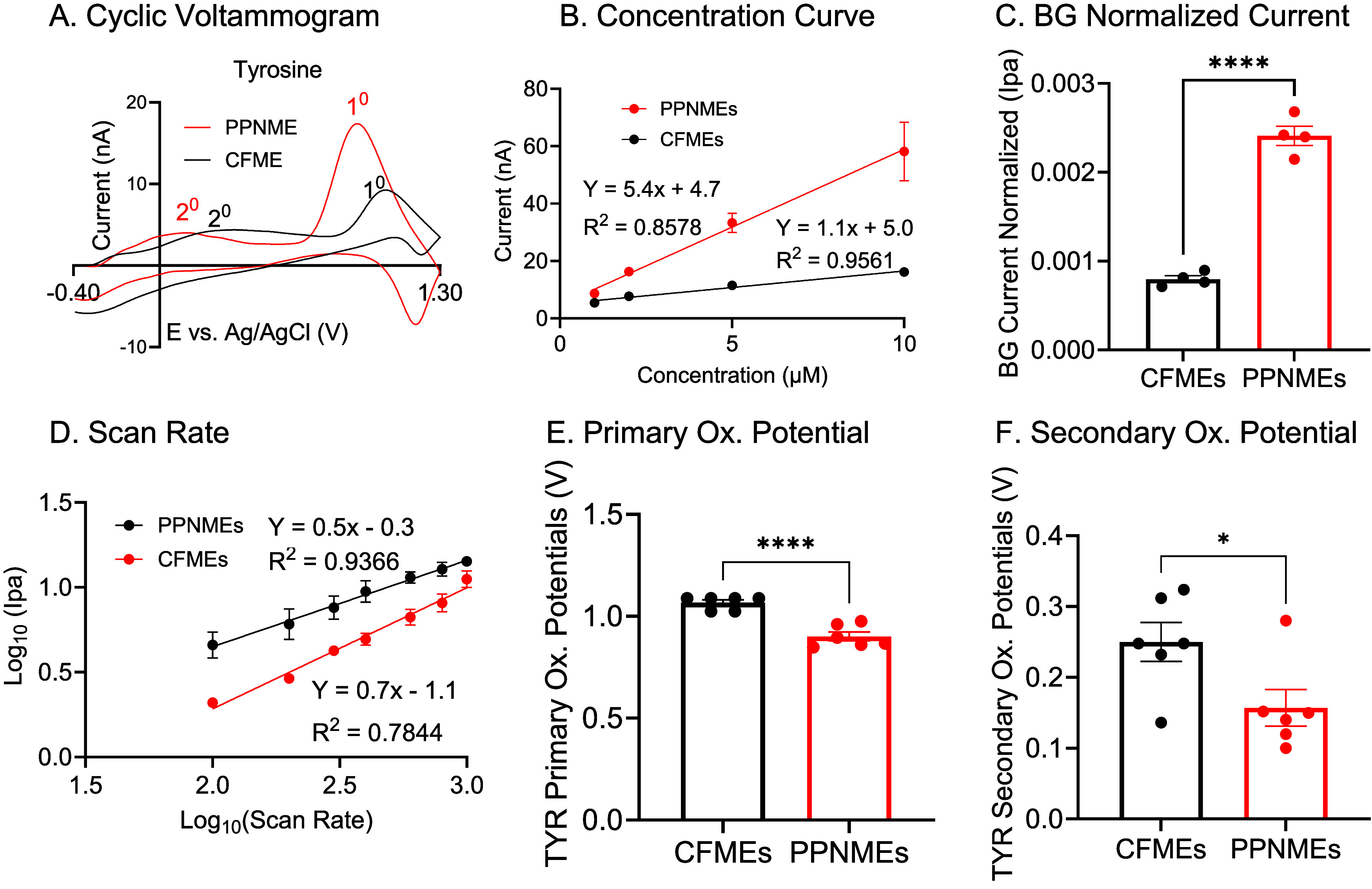
Electrochemical characterization of l-Tyrosine at CFMEs and PPNMEs. (A) CVs of 10 μM Tyr. (B) Tyr sensitivity tests (1–10 μM). Reproducibility analysis yielded an RSD of 16% at 5 μM Trp (PPNME) and 13% for CFME. (C) Background normalized current comparison for 1 μM Tyr (*n* = 4, *t* test, ****p < 0.0001). (D) Tyr scan rate tests (100–1000 V/s). (E) Tyr primary oxidation potentials (*n* = 6, *t* test, ****p < 0.0001). (F) Tyr secondary oxidation potentials (*n* = 6, *t* test, *p = 0.0335). Error bars are SEM.

To assess sensitivity and linearity, we obtained calibration curves for Tyr over the 1–10 μM range (Figure 3B). Both electrodes showed linear current responses, but the sensitivity at PPNMEs (5.4 ± 0.7 nA/μM) was nearly five times higher than at CFMEs (1.1 ± 0.1 nA/μM). This marked increase in sensitivity at PPNMEs is attributable to the same factors discussed for Trp, namely, increased surface area, higher density of adsorption sites, and crevices that momentarily trap the analyte. To ensure that the observed differences were not solely due to increased surface area, we normalized the faradaic currents to background charging currents (Figure 3C). Even after normalization, PPNMEs exhibited 3.1-fold higher current responses for Tyr compared to CFMEs. This provides further evidence that the pyrolyzed parylene surface intrinsically enhances sensitivity, rather than simply increasing the area.

We then performed scan rate studies to determine the mechanism of Tyr detection at each electrode (Figure 3D). For CFMEs, the slope of the log­(current) vs log (scan rate) plot was 0.51, consistent with a diffusion-controlled process. At PPNMEs, the slope increased to 0.71, indicating that Tyr detection is more adsorption-controlled at these electrodes. The increased adsorption is likely due to trapping effects at PPNMEs, where the analyte is momentarily trapped in crevices on the PPNMEs, allowing secondary product oxidation to be more readily detected. Trapping can preconcentrate the analyte, resulting in thin-layer diffusion effects and higher currents.

Finally, we compared the oxidation potentials for Tyr at both electrode types (Figure 3E,F). Both the primary and secondary oxidation potentials were significantly lower at PPNMEs than at CFMEs, supporting the conclusion that the PPNME surface accelerates electron transfer and lowers the energy barrier for Tyr oxidation. The enhanced sensitivity and lower oxidation potentials observed for Tyr at PPNMEs mirror those seen for Trp, suggesting that the benefits of the RTP-polymer modified surface extend to other aromatic amino acids.

### Proposed Oxidative Mechanisms

The oxidative mechanisms of tryptophan and tyrosine are summarized in Scheme 1A,B, respectively. The initial oxidation of tryptophan involves the loss of 2 electrons and protons, followed by hydration, resulting in the formation of a carbonyl group on the indole ring. This leads to the primary oxidized product, oxindolylalanine (1°).^18,22,28^ Further oxidation and rearrangement of the primary product yield secondary products. The secondary product (2°) is dioxindolylalanine, formed through further cleavage and deprotonation steps. The tertiary product (3°) is trioxindolylalanine or other ring-open structures, which arise from continued oxidative cleavage and hydration of the indole ring. These oxidized products reflect the complexity of tryptophan oxidation, often resulting in a mixture of compounds depending on the oxidative environment. Tyrosine, a phenolic amino acid, undergoes a similar sequence of oxidative transformations. The primary oxidation involves the loss of an electron and a proton to form a primary oxidized product, tyrosyl radical (1°), which then undergoes hydration to yield the secondary product (2°) commonly identified as dopaquinone or L-DOPA.^29^

PPNMEs showed enhanced secondary and tertiary peaks due to the trapping of intermediates near the surface, resulting in higher currents for these products. These trapping effects have been extensively described at CNY yarn electrodes and other carbon nanoelectrodes for dopamine and catecholamines,^10,27,30^ But have not been tested for peptides.

If the electrode surface is rough or has crevices that trap analyte, oxidized intermediates and products are retained near the surface for an extended period and are more likely to undergo further oxidation.^3,31–34^ The PPNMEs have more crevices than the CFMEs, increasing the likelihood of detecting secondary and tertiary oxidation products.^10^ Furthermore, PPNMEs have more oxygen functional groups, which also promote adsorption.^10^ This is the first time trapping effects have been explored for electroactive peptides, and the PPNMEs show that peptides exhibit enhanced secondary and tertiary peaks at electrodes with increased surface roughness.

### Comparative Performance of Trp and Tyr at PPNMEs

The RTP-parylene electrodes significantly enhanced the electrochemical detection of both Trp and Tyr compared to conventional CFMEs. With PPNMEs, sensitivity increased over 3-fold for tryptophan and nearly 5-fold for tyrosine, alongside notable shifts to lower oxidation potentials.

Increases in sensitivity are similar to those reported at other carbon nanomaterial electrodes, such as carbon nanotube (CNT)^35^ and graphene-modified electrodes,^36^ which consistently show multifold enhancements in sensitivity for small biomolecules like dopamine and serotonin, due to increased porous surface and improved electron transfer properties. The mechanism underlying these enhancements is widely attributed to both an increased effective electrode surface, the rich defect sites and oxygen functional groups, which promote the neurochemical adsorption of cationic neurotransmitters^37,38^ Reports on mechanisms of peptide detection at carbon-based electrodes are rare; however, the observed sensitivity and adsorption-controlled kinetics in our system closely resemble those documented for dopamine and related analytes,^35,36,39^ suggesting that analogous mechanisms are responsible for the improved performance seen here.

The increased porous structure, confirmed by SEM analysis, facilitates trapping in crevices and more frequent analyte–surface interactions, thus elevating the overall current response. Scan rate analysis further highlights the distinct electrochemical behavior of Trp and Tyr at PPNMEs. Both amino acids exhibit slopes greater than 0.7 on log–log plots of peak current versus scan rate, indicating a shift toward adsorption-controlled kinetics, unlike the diffusion-controlled pattern observed with CFMEs. This behavior suggests that the PPNME surface promotes preconcentration of aromatic amino acids via π–π stacking or electrostatic interactions, which enhances the sensitivity for Trp and Tyr. Additionally, the thin-layer diffusion effect caused by increased surface roughness promotes an adsorption-like behavior, increasing the trapping and resulting in enhanced secondary peaks.^27,40,41^ Furthermore, voltammograms for both analytes at PPNMEs display additional or more resolved peaks, likely due to the complex heterogeneous nature and higher roughness of the PPN surface.

### Electrochemical Detection of Gonadotropin-Releasing Hormone (GnRH)

We next evaluated PPNMEs for the detection of a more complex analyte: gonadotropin-releasing hormone (GnRH). GnRH is a decapeptide neurohormone that contains both tryptophan and tyrosine residues. CVs were recorded for 2 μM GnRH at both CFMEs and PPNMEs (Figure 4A) with the dopamine waveform. At CFMEs, GnRH had two oxidation peaks: a primary peak at 1.05 V (13 nA) and a secondary peak at 0.45 V (3.6 nA). At PPNMEs, the primary oxidation peak was observed at a lower potential (0.96 V), and the current peak was higher at 19.5 nA. A secondary peak was also present at 0.42 V (10 nA). The lower oxidation potential and increase in current at PPNMEs are consistent with the trends observed for Trp and Tyr, indicating that the RTP-parylene surface also enhances the detection of GnRH.

**Figure 4:**
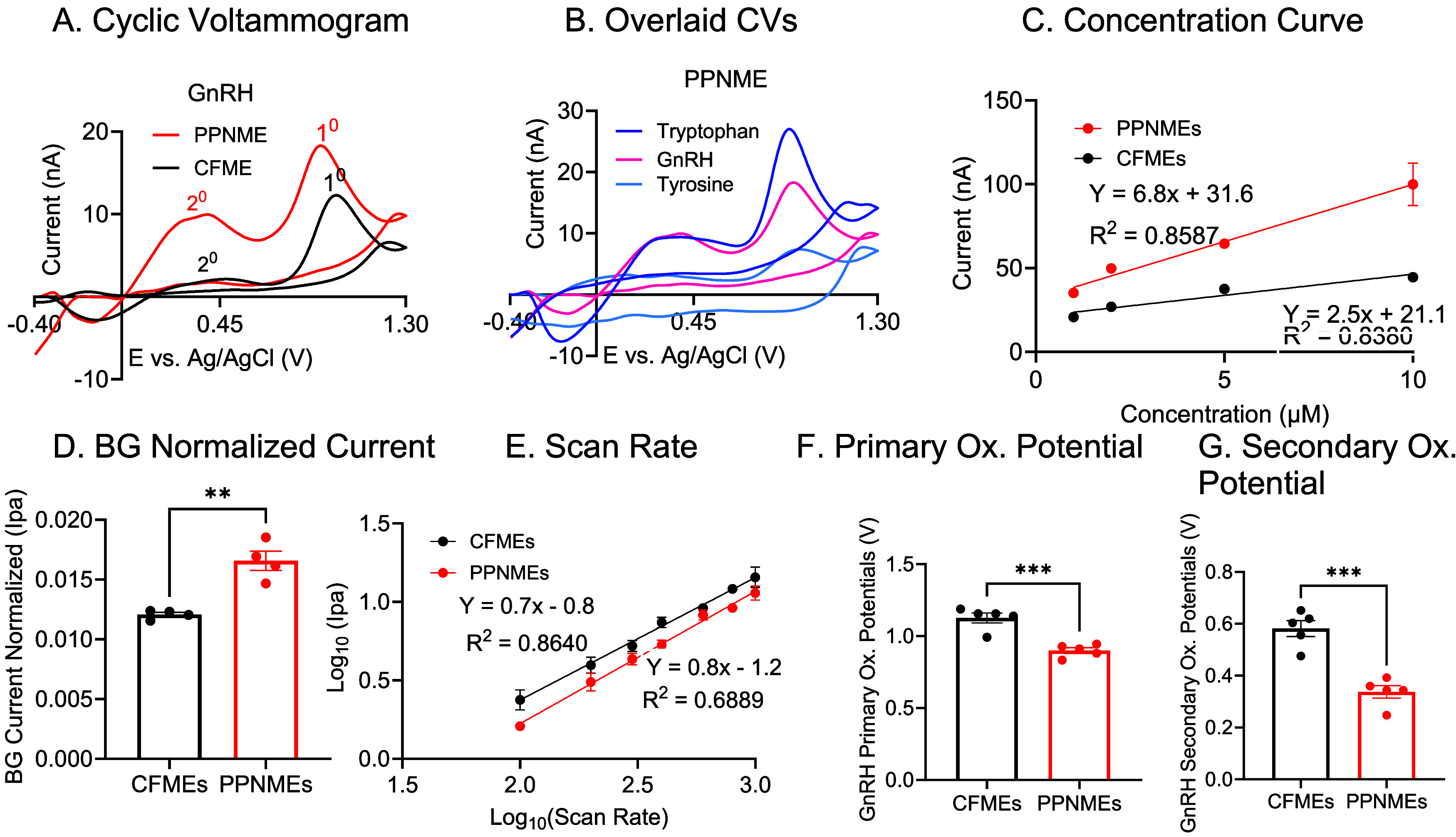
Electrochemical characterization of GnRH at CFMEs and PPNMEs. (A) CVs of 2 μM GnRH. (B) CVs of 2 μM Trp, Tyr, and GnRH. (C) GnRH sensitivity tests (1–10 μM). The PPNME showed an RSD of 12% at 5 μM GnRH and 17% for CFME. (D) Background normalized current comparison for 1 μM GnRH (*n* = 4, *t* test, ****p < 0.0001). (E) GnRH scan rate tests (100–1000 V/s). (F) GnRH primary oxidation potentials (*n* = 5, *t* test, ***p = 0.0002). (G) GnRH secondary oxidation potentials (*n* = 5, *t* test, ***p = 0.0004). (Error bars are SEM).

To directly compare the electrochemical signatures of Trp, Tyr, and GnRH, we overlaid their CVs at PPNMEs (Figure 4B). The primary oxidation peaks for all three analytes occurred at similar potentials, suggesting that the oxidation of GnRH is dominated by its Trp and Tyr residues. The voltammetric response of GnRH closely mirrors that of Trp. Given the higher sensitivity of the electrode for Trp over Tyr, it is not surprising that the Trp residue serves as the primary site of electrooxidation within the intact peptide. Although GnRH contains histidine, histidine oxidizes near +1.3 V under our FSCV conditions, which is outside the potential window where GnRH, Trp, and Tyr oxidize. The histidine CV (Figure S2) confirms that histidine does not contribute to the GnRH oxidation signal. Calibration curves for GnRH were obtained over the 1–10 μM range (Figure 4C). PPNMEs exhibit a sensitivity of 6.8 ± 0.3 nA/μM, which was 2.7 times higher than that of CFMEs (2.5 ± 0.3 nA/μM). This enhanced sensitivity is consistent with our observations for individual amino acids and further underscores the advantages of the PPNME surface for detecting peptides containing electroactive residues.

To account for differences in surface area, we normalized the faradaic currents to background charging currents (Figure 4D). PPNMEs had significantly higher normalized currents for GnRH, indicating that the increased sensitivity is not solely a function of increased surface area, but also reflects the intrinsic properties of the surface. Scan rate studies provided additional insight into the detection mechanism (Figure 4E). For CFMEs, the slope of the log­(current) vs log (scan rate) plot was 0.7, indicating a more diffusion-controlled process. At PPNMEs, the slope increased to 0.80, indicating that GnRH detection is more adsorption-controlled at these electrodes. Again, this behavior mirrors that of the individual amino acids and is likely due to increased crevices, trapping effects, and surface functional groups that enhance adsorption.

Finally, we compared the oxidation potentials of GnRH (Figure 4F,G). PPNMEs exhibited lower primary and secondary oxidation potentials for GnRH, like the values observed for Trp, suggesting that the Trp residue within the peptide is primarily responsible for its electrochemical behavior. The enhanced sensitivity and lower oxidation potentials observed for GnRH at PPNMEs are consistent with the trends seen for Trp and Tyr; thus, the RTP-parylene surface is particularly effective for detecting peptides containing electroactive aromatic residues. The thin-layer cell effects of the crevices at PPNMEs further enhance sensitivity and enhance electron transfer by promoting analyte preconcentration at the electrode surface.

The PPNMEs demonstrated good reproducibility across all analytes, with relative standard deviations (RSDs) at 5 μM of 6% for Trp, 16% for Tyr, and 12% for GnRH (n = 4). These values indicate consistent electrode performance and reliable analytical behavior across independently fabricated devices. The CFMEs also showed good reproducibility, with RSDs at 5 μM of 16% for Trp, 13% for Tyr, and 17% for GnRH (*n* = 4). These results further confirm dependable analytical responses across separate electrode preparations.

### Detection of GnRH in Brain Tissue

PPNMEs were tested for GnRH detection in mouse brain slices. First, the electrode was inserted 75 μm into the slice, and 100 μM GnRH was locally applied using a nanoliter injector. The background-subtracted CV displayed a peptide oxidation peak at 1.20 V with a current of 12 nA (Figure 5A). The oxidation peak occurred at a slightly more positive potential in the brain slice (1.2 ± 0.04 V) compared with the in vitro measurements (1.0 ± 0.07 V). While the observed faradaic response confirms an oxidative process, the peak appears during the reverse scan from the switching potential back to the holding potential. This behavior is most likely due to reduced electron-transfer kinetics for electrodes operating within tissue, which is consistent with the altered chemical environment surrounding the peptide.^14^ Additional features appearing at approximately 0.1 and 0.12 V were observed during fluid application; these arise from interference peaks associated with changes in double-layer charging at the electrode surface rather than peptide oxidation. Such interference is expected in tissue due to changes in local ionic composition and capacitive currents during peptide application. Previous FSCV studies of GnRH in brain slices reported similar distortions in CV shape, often detecting only a single reverse peak, highlighting the well-known effect of tissue impedance, adsorption, and limited mass transport on peptide voltammograms.^14^ Importantly, unlike the earlier work that required a modified waveform beginning at 0.5 V to minimize fouling, PPNMEs allowed GnRH detection using the standard dopamine waveform.

**Figure 5:**
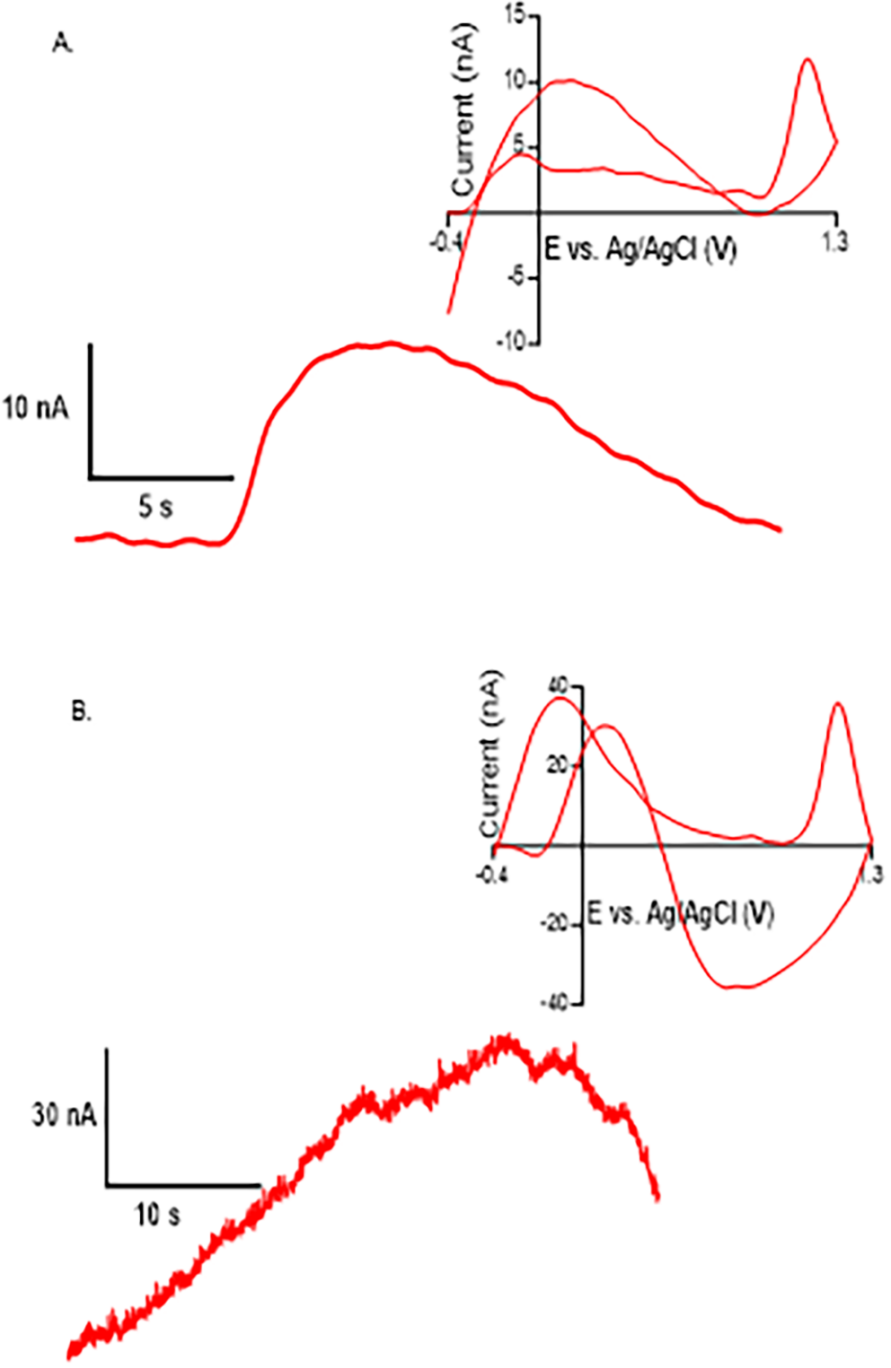
GnRH detection in a brain slice at PPNME electrode. (A) I vs T trace of Puffed GnRH via a nanoliter injector near the working electrode in a wild-type mouse brain slice. Inset: CV of puffed-on GnRH. (B) I vs T trace of spontaneous GnRH in wild-type mouse brain slice of the median eminence. Inset: CV of spontaneous GnRH release.

To further assess biological applicability, PPNMEs were then used to record spontaneous, endogenous GnRH release in slices containing the median eminence (ME). The electrode was inserted 50–75 μm into the tissue, and voltammograms were collected over 5 min. Spontaneous GnRH transients were observed and produced a peak with a concentration of 0.65 μM at 1.20 V (Figure 5B), accompanied by the same interference peaks (∼ −0.1 and 0.12 V) seen during the puff-on experiment. In the future, we could change waveforms to eliminate these lower peaks, which we did in the previous paper.^14^ The similarity between the puffed-on and spontaneous tissue signals, including both the interference features and the main oxidation peak, strongly supports the conclusion that the 1.2 V peak reflects GnRH detection under biological conditions. These data demonstrate that PPNMEs can detect both exogenous and endogenous GnRH release in tissue, confirming their suitability for neuropeptide measurements.

## Conclusions

This study demonstrates that PPNMEs have significantly higher sensitivity compared to CFMEs for detecting tryptophan, tyrosine, and GnRH, with up to a 5-fold improvement in current response and lower oxidation potentials. SEM imaging revealed that PPNMEs possess a highly porous nanostructured surface, which enables adsorption-controlled electrochemical processes and contributes to improved analyte preconcentration. The detection of multiple oxidation peaks, particularly at PPNMEs, is consistent with known oxidative pathways of aromatic amino acids and is due to trapping effects caused by increased crevices and surface roughness. PPNMEs effectively detected GnRH, a neuropeptide containing Trp and Tyr, with enhanced current responses, demonstrating that these electrodes can be used to monitor more complex biomolecules in neural environments. Finally, in situ detection of GnRH in mouse brain tissue using PPNMEs highlights the platform’s real-world applicability. Both puffed-on GnRH and spontaneous endogenous GnRH release produced detectable oxidation signals near 1.2 V, further confirming that PPNMEs can monitor peptide dynamics directly within tissue. Despite minor shifts in potential, presumably due to tissue matrix effects and interference from other biomolecules, the oxidation peak of the peptide was detectable, demonstrating sensitivity and temporal resolution in a complex biological environment. PPNMEs offer a new method for electrode design and expand its application for monitoring various neuroactive peptides and small molecules in complex biological systems.

## Supplementary Material



## Notes

Figure S1: Background cyclic voltammograms (CVs) of CFME and PPNME; Figure S2: Cyclic voltammogram of histidine at PPNME (PDF)
