# Supplementary material for: Pyrolyzed Parylene Electrodes for Detection of Tryptophan, Tyrosine, and Gonadotropin-Releasing Hormone
Source: ACS Meas Sci Au. 2025 Dec 26;6(1):214–23. doi: 10.1021/acsmeasuresciau.5c00165 (PMC12921596; doi:10.1021/acsmeasuresciau.5c00165)
Supplement: Supplementary file 1 [file tg5c00165_si_001.pdf]

Supporting Information

Pyrolyzed Parylene Electrodes for detection of tryptophan, tyrosine, and gonadotropin-releasing hormone

Faith Eyimegwu [a, †], He Zhao [a], Kailash Shrestha [a], Dayana Surendran [a], Nickolay V. Lavrik [b], B. Jill Venton [a, *]

[a] Department of Chemistry, University of Virginia, Charlottesville, Virginia, 22901, USA

Office #: PLSB108

Phone: (434) 243-2132

Email: bjv2n@virginia.edu

*Corresponding author

[b] Center for Nanophase Materials Sciences, Oak Ridge National Lab, Tennessee, 37831, USA

Table of Contents:

Figure S1: Background Cyclic Voltammograms (CVs) of CFME and PPNME

Figure S2: Cyclic Voltammogram of histidine at PPNME

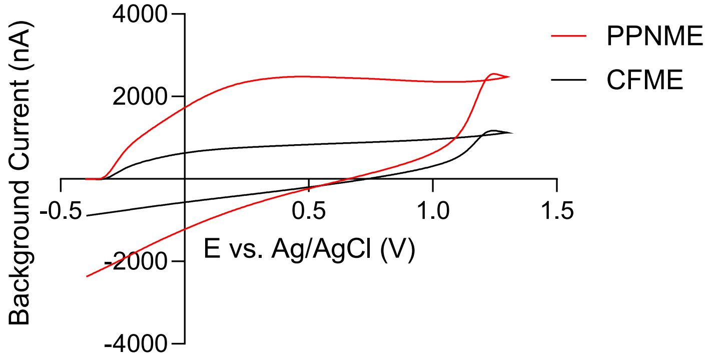

S1. Background Cyclic Voltammograms (CVs) of CFME and PPNME

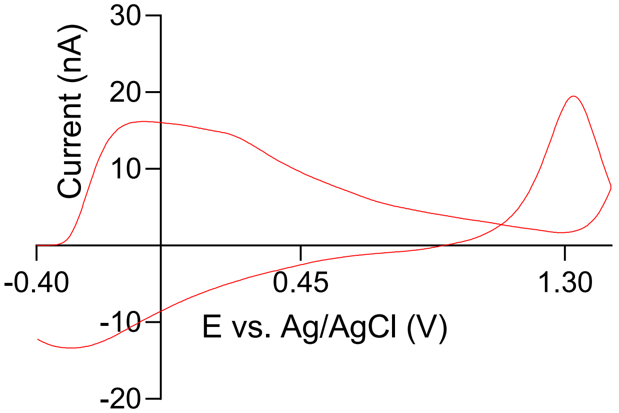

S2. Cyclic Voltammogram of histidine at PPNME. Oxidation potential is at 1.3V, which is above the oxidation potential of the peptide
